# Research progress of circulating non-coding RNA in diagnosis and treatment of hepatocellular carcinoma

**DOI:** 10.3389/fonc.2023.1204715

**Published:** 2023-07-20

**Authors:** Junqi You, Haoming Xia, Ziyue Huang, Risheng He, Xudong Zhao, Jiali Chen, Sidi Liu, Yi Xu, Yunfu Cui

**Affiliations:** ^1^ Department of Pancreatobiliary Surgery, The Second Affiliated Hospital of Harbin Medical University, Harbin, Heilongjiang, China; ^2^ Department of Anesthesiology, The Second Affiliated Hospital of Harbin Medical University, Harbin, Heilongjiang, China

**Keywords:** circulating noncoding RNA, hepatocellular carcinoma, biomarker, diagnosis, treatment

## Abstract

Hepatocellular carcinoma (HCC) is a highly malignant tumor that carries a significant risk of morbidity and mortality. This type of cancer is prevalent in Asia due to the widespread presence of risk factors. Unfortunately, HCC often goes undetected until it has reached an advanced stage, making early detection and treatment critical for better outcomes. Alpha-fetoprotein (AFP) is commonly used in clinical practice for diagnosing HCC, but its sensitivity and specificity are limited. While surgery and liver transplantation are the main radical treatments, drug therapy and local interventions are better options for patients with advanced HCC. Accurately assessing treatment efficacy and adjusting plans in a timely manner can significantly improve the prognosis of HCC. Non-coding RNA gene transcription products cannot participate in protein production, but they can regulate gene expression and protein function through the regulation of transcription and translation processes. These non-coding RNAs have been found to be associated with tumor development in various types of tumors. Noncoding RNA released by tumor or blood cells can circulate in the blood and serve as a biomarker for diagnosis, prognosis, and efficacy assessment. This article explores the unique role of circulating noncoding RNA in HCC from various perspectives.

## 1 Introduction

Hepatocellular carcinoma (HCC) is the most prevalent type of cancer that originates in the liver. It ranks fifth in terms of overall cancer occurrences worldwide and is second in terms of fatality among malignant tumors (1). Unfortunately, the incidence of HCC continues to increase as its primary risk factors, including non-alcoholic steatohepatitis, hepatitis B virus infection, and hepatitis C virus infection, remain widespread (2). While current research is focused on examining the molecular mechanism of HCC, there has been no substantial improvement in the prognosis of advanced cases. This is due to the aggressive nature of HCC and its tendency to recur, resulting in an overall survival rate that falls short of satisfactory (3). Early diagnosis plays a critical role in improving the survival time and rate of patients with HCC. Currently, the primary means of clinical diagnosis includes assessing serum alpha-fetoprotein (AFP) levels and utilizing imaging examinations like liver ultrasound, CT, and MRI (4). Although alpha-fetoprotein is a serum biomarker, its specificity is not strong enough. Therefore, current research aims to discover a biomarker that boasts high diagnostic accuracy and specificity. Drug therapy has limited effectiveness, and for patients with early-stage HCC, surgical resection or liver transplantation remains a promising standard of treatment (5). At the same time, in addition to drug therapy and surgery, local radiofrequency therapy and transcatheter arterial chemoembolization (TACE) have gradually been accepted clinically as treatment methods for HCC. At present, drug resistance in HCC has gradually emerged, which has become the main bottleneck limiting the effect of drugs. By continuously improving these treatments, patients with HCC can attain a more favorable prognosis.

Biomarkers play an important role in the field of oncology. For example, alpha-fetoprotein (AFP) is a widely accepted biomarker for the detection of HCC, while CA-199 is sensitive to pancreatic cancer (6), and carcinoembryonic antigen (CEA) is commonly used for colon cancer patients (7). These are just a few examples of the many biomarkers in use today. Biomarkers can: 1) classify patients according to risk; 2) Diagnosis and detection of disease development; 3) Effectively derive patient prognosis and adjust treatment (8). Biomarker tests known as “liquid biopsies” are extracted from body fluids such as blood and urine. This method is less invasive and less painful compared to traditional biopsies. Additionally, it provides relatively good insight into the heterogeneity of tumor molecules and is highly sensitive (9). This liquid biopsy offers numerous advantages over tissue biopsy. Firstly, it inflicts less trauma, making it more appealing for patients. Additionally, it is convenient and fast, offering a simpler and quicker alternative. Moreover, this method is much more accurate and precise. Finally, it also reduces the likelihood of tumor metastasis.

## 2 Non-coding RNA

**Figure 1:**
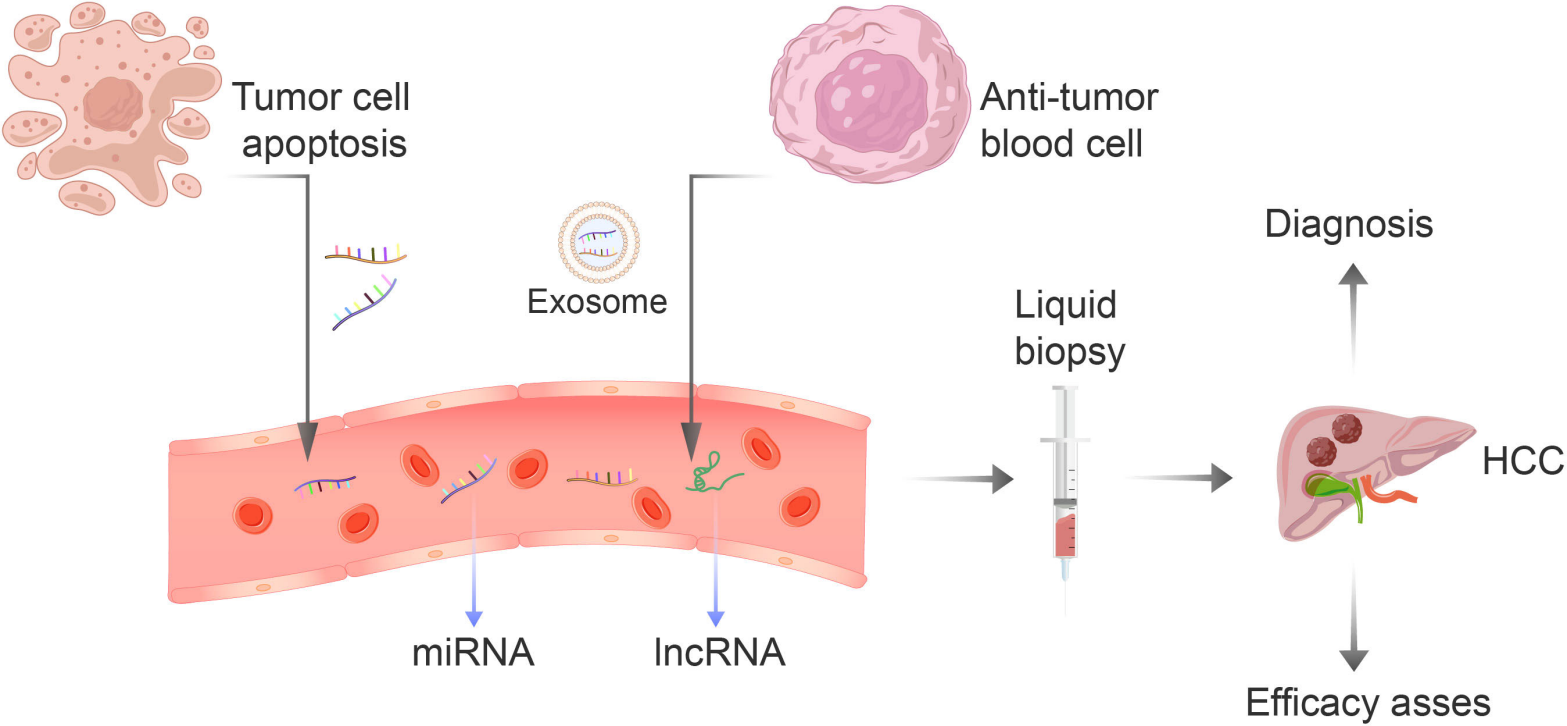
The non-coding RNA present in blood is primarily derived from two sources: the apoptosis of tumor cells or the secretion of blood cells that have anti-tumor properties. This RNA includes miRNA-122, let-7 family, miRNA-34a, and lncRNA. The liquid biopsy technique is an efficient, convenient, and non-invasive method for early detection and evaluation of the effectiveness of treatment for liver cancer.

Since the discovery of non-coding RNA in the 1990s, researchers have continuously delved deeper into its study, leading to a greater understanding of how gene expression is regulated (10). Non-coding RNA is a byproduct of gene transcription, which cannot be used for protein production. Nonetheless, it has the ability to regulate gene expression and protein function by controlling both transcription and translation processes (11). Through these basic biological functions, it participates in the process of cell growth, migration, autophagy, apoptosis and differentiation, and is widely involved in biological behavior (12). Non-coding RNAs are implicated in the development of numerous diseases, including cancer, heart disease, and immune disorders. As a result, some researchers are now exploring their potential as biomarkers for early diagnosis and treatment evaluation in patients with hepatocellular carcinoma. However, the use of non-coding RNA for tissue assessment may cause discomfort to patients as it requires invasive procedures, unlike using serum AFP. The concentration level of circulating non-coding RNA is relatively stable because of the protection provided by exosomes, making it an effective tool for distinguishing tumor patients from normal individuals (13). Circulating non-coding RNAs primarily result from the discharge of intracellular non-coding RNAs into the bloodstream due to tissue damage or from the discharge of cells as exosomes (14) (**Figure 1**). The utilization of circulating noncoding RNAs as a means of identifying biomarkers has shown considerable promise in various types of cancer, including lung, prostate, and gastric cancers (15). Non-coding RNA found in the circulation offers significant potential as a non-invasive biomarker for HCC, leaving ample room for research opportunities. Employing non-coding RNA as a biomarker could bring considerable enhancements to the diagnosis, prognosis, and treatment efficacy of HCC. The main components of circulating ncRNA are circulating microRNA and circulating long non-coding RNA (lncRNA). Unfortunately, there are currently limited studies conducted on circulating circular RNA (circRNA). Additionally, no obvious correlation with tumors has been found, so it will not be discussed further in this paper. Therefore, this article will primarily focus on the first two aspects: circulating microRNA and circulating lncRNA.

## 3 Circulating microRNA

MicroRNA is a type of small non-coding RNA that consists of approximately 18-24 nucleotides in length. Once matured, microRNA joins the RNA-induced silencing complex located in the cytoplasm. From there, it combines with the 3’untranslated region (3’-UTR) of the mRNA to facilitate degradation and translational repression. The primary goal of this process is to regulate gene expression (16, 17). Abnormal regulation of microRNA often leads to the development of tumors. MicroRNA can bind to proteins present in different body fluids, or it can be enveloped in bilayer lipids to attain a stable state. The stability of microRNA is the highest when it is situated in exosomes. Thus, to assess its potential as a biomarker, basic quantitative polymerase chain reaction (qPCR) and other methods can be employed (18). The microRNAs that only exist in serum are derived from non-blood cells, some of which are derived from tumor cell apoptosis or secretion (19). Most of the microRNAs in the circulation mainly come from blood cells that exert anti-tumor functions (20). Furthermore, the genetic and molecular characteristics of a tumor can be revealed by analyzing circulating microRNA. This method is especially effective at detecting the changes that occur in response to tumors. In fact, the levels of serum microRNA are typically 1.6 times higher in tumor patients compared to those of healthy individuals (21). Abnormal expression of miRNA in patients with HCC is closely related to the occurrence and development of tumors, and the let-7 family (22) and miRNA-122 expression are downregulated widely reported in HCC (23), and miRNA-221, miRNA-222 and miRNA-224 are upregulated (24, 25). According to research, miRNA-122, miRNA-34a, and miRNA-196a-5p are known to suppress tumors, while the let-7 family of miRNAs have been found to promote cancer growth. Compared to other malignant tumors, the abnormal miRNA profile of HCC is more distinct in terms of diagnosis, treatment, and prognosis (26). Incorporating miRNA as a biomarker and utilizing it in clinical practice can significantly enhance clinicians’ ability to gain a more precise understanding of their patients’ conditions, ultimately leading to more accurate medical decisions.

### 3.1 Circulating microRNAs and HCC diagnosis

**Figure 2:**
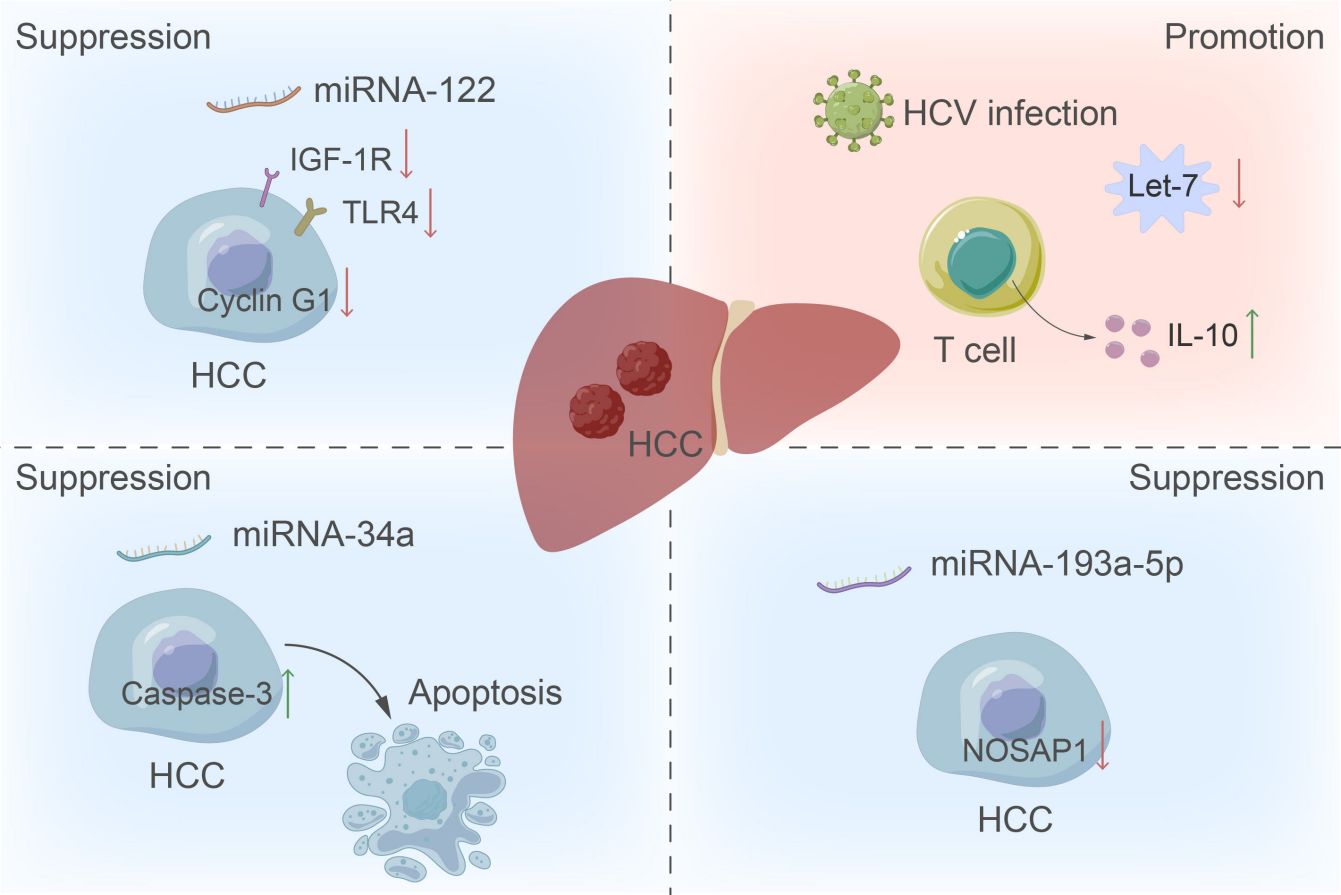
The role of miRNA in the development of HCC. The up-regulation of miRNA-122 inhibits liver cancer by inhibiting cyclin G1, the insulin-like growth factor 1 receptor pathway, and the expression of toll-like receptor 4 in tumor cells. Conversely, downregulation of the let-7 family increases T cell production of IL-10, providing the virus with a survival advantage by manipulating the host immune response and promoting tumor formation. Additionally, miRNA-34a functions as a tumor suppressor by inducing apoptosis through increased activity of caspase-3.

Currently, the incidence of HCC is consistently increasing each year. Due to its deceptive symptoms and rapid progression, it is commonly detected in the advanced stages of tumor development during diagnosis (27). At present, the primary method for early screening of liver cancer is measuring the serum AFP levels. However, this method’s low sensitivity has led to debates surrounding its diagnostic efficacy (28). The AFP level is found to be elevated not only in patients with HCC, but also in those with chronic hepatitis B and hepatitis C infections (29). Recent studies have suggested that the combination of miRNA and AFP could markedly enhance early detection of HCC by improving both sensitivity and specificity (30). Among them, many studies have been published on the role of circulating miRNA-122 (31), let-7 family (32) and other miRNAs in the diagnosis of HCC (**Figure 2**).

#### 3.1.1 miRNA-122

miRNA-122 is a major microRNA in the liver, accounting for approximately 70% of all liver microRNAs (33), and is involved in the development and pathogenesis of HCC (34). The expression of miRNA-122 is regulated by factors such as enhancer-binding protein α, hepatocyte nuclear factor (HNF) 1α, HNF3β, and HNF4α (35),which explains why miRNA-122 is a liver-specific microRNA. miRNA-122 can inhibit the expression of p53 by down-regulating the cell cycle G1-p53 complex to inhibit the RNA replication of hepatitis B virus, and hepatitis B virus can regulate miRNA-122, reduce its expression to promote virus infection, and eventually lead to tumorigenesis (36). Previous studies have shown that the absence of miRNA-122 will promote liver steatosis and liver fibrosis, and the reduction of miRNA-122 levels has been observed in people with non-alcoholic steatohepatitis and liver cirrhosis (37). Liver tumors appeared successively in mice knocked out of the miRNA-122 gene, and liver tumors in mice were suppressed when microRNA levels were restored (38). miRNA-122 exerts its tumor suppressive effect by inhibiting cyclin G1, inhibiting the insulin-like growth factor 1 receptor pathway, inhibiting the expression of toll-like receptor 4 in tumor cells, etc. (37).

Serum miRNA-122 levels in the HCC group were found to be significantly elevated in early-stage HCC patients compared with healthy controls (31). When miR-122-5p is detected in the circulation, it indicates the early occurrence of liver cancer (39). However, there are similar problems with AFP in the diagnosis of liver cancer. At present, only HCC patients and healthy people are compared, and the serum level in non-HCC patients such as chronic hepatitis B virus infection and liver cirrhosis is still unclear. A recent meta-analysis study showed that (40), the level of miRNA-122 in serum was significantly different between patients with HCC and healthy people or patients with chronic hepatitis B virus infection. However, for patients with liver cirrhosis, the sensitivity of judging whether there is HCC is not high, and auxiliary examinations such as imaging studies are still needed. And in a survey of European population, it was found that serum miRNA-122 was significantly increased in patients with acute liver injury, and it was positively correlated with transaminase levels and alpha-fetoprotein levels (41). Although the sensitivity of miRNA-122 in the diagnosis of liver cancer is better than that of AFP (42), it still has its shortcomings. Some researchers proposed to analyze the binding of circulating miRNA-122 to telomerase reverse transcriptase in circulating cell DNA to evaluate its diagnostic efficacy as a biomarker. The differential diagnosis of HCC, cirrhosis and non- HCC is the highest (43). And miRNA-122 is a kind of tumor suppressor factor, and the serum concentration rises when HCC occurs, which may be secreted from blood cells that play an anti-tumor effect.

#### 3.1.2 let-7 family

The let-7 family consists of 12 miRNAs that function as microRNAs. They play a crucial role in negatively regulating oncogenes and cell cycle factors (44). Relevant studies have pointed out that the circulating let-7 family can be mainly divided into three clusters according to clustering, and let7 b/c/g are the representatives of the three clusters (45). The let-7 family mainly targets hepatitis C virus infection, and interferon α increases the production of let-7s through the signaling pathway to inhibit the replication of hepatitis C virus (46). Moreover, the level of let-7 in HCC tissues caused by hepatitis C virus infection is lower than that in healthy people, and its circulating level is also negatively correlated with the degree of liver cirrhosis (47), indicating that the decreased level of let-7 family in the circulation should be related to HCV infection. Downregulation of the let-7 family leads to increased production of IL-10 by T cells, which in turn confers an important survival advantage to the virus by manipulating the host immune response (48). Subsequent studies have also found that the level of let-7i in the circulation after antiviral therapy can be used as a biomarker for the progression of HCC (32). Circulating miRNAs have good diagnostic efficacy for hepatitis C viral HCC developed from liver cirrhosis and chronic hepatitis C virus, and when used as non-invasive disease biomarkers together with alpha-fetoprotein, the diagnostic efficacy have significantly improved (49). By detecting the level of the let-7 family in the circulation and determining the presence of hepatitis C virus infection, it is possible to identify the condition of patients with hepatitis C virus infection in a timely manner and detect HCC at an early stage.

#### 3.1.3 miRNA-34a

MiRNA-34a has been confirmed as a tumor suppressor (50), that is, patients with tumors with high expression levels of miRNA-34a have a better prognosis. In hepatocellular carcinoma, abnormal expression of miRNA-34a promotes tumor proliferation and metastasis through cell cycle and p53 signaling pathway (51). Recent studies have found that miRNA-34a can significantly downregulate the expression of transcriptional activators E2F1 and E2F3, both of which are upregulated in HCC patients and are associated with the degree of malignancy of tumors (52). However, miRNA-34a induces apoptosis through caspase-3 (53). It was indeed observed in subsequent *in vitro* experiments that miRNA-34a significantly increased the activity of caspase-3 (52). The presence of suppressed tumor suppressors in the tissues of HCC patients should also be at lower levels secreted by tumor cells into the circulation via exosomes. miRNA-34a is a specific and sensitive indicator for the diagnosis of hepatocellular carcinoma (54). After investigation, it was found that the serum miRNA-34a level of patients with HCC was much lower than that of healthy people, and the circulating level of patients before surgery was also lower than that of patients after surgery. Moreover, the decrease of miRNA-34a level in the circulation is related to the clinical characteristics of the tumor, such as TNM stage, vascular invasion and lymph node metastasis, etc. (55) Upregulation of miR-34a-5p levels in serum can predict the onset of cirrhosis (56), which can also point to early changes in HCC (39).

#### 3.1.4 Other microRNAs

Furthermore, in addition to the aforementioned three microRNAs, there are numerous additional microRNAs that are currently being studied for their potential in aiding the diagnosis of HCC. For instance, the miRNA-193a-5p has shown promise as a tumor suppressor, as evidenced by its reduced expression in patients with HCC (57). This microRNA appears to hinder tumor growth by lowering levels of nucleoli and spindle-associated protein 1, and its low expression levels are significantly linked to unfavorable prognoses (58). Through comparison of the circulating miRNA-193a-5p levels of postoperative HCC patients with those of healthy individuals, it was discovered that patients with HCC exhibited elevated circulating levels of this particular miRNA. Moreover, those who had higher levels of miRNA-193a-5p prior to surgery had generally poorer survival outcomes. This increased level of miRNA-193a-5p in circulation is likely attributable to tumor cell apoptosis, which results in greater secretion of this miRNA into the bloodstream (59).. In patients with hepatocellular carcinoma, the level of miRNA-223-3p in circulation is significantly reduced, and the diagnostic accuracy of alpha-fetoprotein can reach 100% for intermediate and advanced hepatocellular carcinoma. miRNA-223-3p is an independent prognostic factor for patients with HCC (60). Research indicates that the detection of miR-100-5p, miR-34a-5p, and miR-365-5p in the bloodstream can signify initial alterations prior to the development of HCC (39). In this study, we found that serum miRNA-16 levels were significantly higher in patients with early-stage HCC compared to the control group, after amplifying the circulating samples. This was confirmed by ROC curves, which showed that miRNA-16 had high diagnostic accuracy. Furthermore, analyzing miRNA-16 and alpha-fetoprotein together could further increase its accuracy (42). In this study, we discovered that miRNA-518d-5p expression levels were elevated in both tumor and healthy individuals, as well as in both tumor and non-tumorous specimens of HCC patients. Furthermore, we observed that higher levels of this miRNA in serum samples were correlated with poorer treatment outcomes and shorter treatment times (61). Elevated levels of circulating MiRNA-107 can indicate the early stages of HCC development and have been linked to a poorer prognosis (62).

### 3.2 Circulating microRNAs and HCC efficacy assessment

Currently, there are three main approaches used to treat HCC: drug treatment, surgical treatment, and local interventional treatment. Drug treatment options include chemotherapy, targeted therapy, and immunotherapy. Surgical treatment encompasses radical resection and liver transplantation. On the other hand, local intervention mainly employs radiofrequency therapy and hepatic arterial chemoembolization as its mainstay procedures. Although treatments exist for hepatocellular carcinoma (HCC), these options are primarily effective for patients in the early stage of the disease, which only represents a small fraction of HCC cases (63). Unfortunately, HCC is notorious for its inconspicuous symptoms, and it is typically diagnosed when the tumor has reached an advanced stage. Consequently, surgical treatment offers only limited benefits. As such, drug therapy and local interventional procedures are the predominant methods of treating patients with advanced HCC (64). Hepatocellular carcinoma exhibits heterogeneity both between and within individuals. The former is largely attributable to differences in risk factors and genomic environments, while the latter can be attributed to clonal evolution of cancer cells (65, 66). Heterogeneity can have varying causes, all of which can lead to decreased effectiveness of targeted drugs. A major hurdle in drug treatment is the emergence of drug resistance, which greatly limits efficacy. Unfortunately, there is a current lack of biomarkers that are sensitive to drug resistance. Despite being a potentially effective treatment for HCC, local interventional therapy is not consistently successful in cases of heterogeneous HCC. Consequently, surgeons require a biomarker that can aid in evaluating a patient’s response to treatment in order to address this pressing issue. MicroRNAs can reflect some characteristics of tumors (67), and it is generally believed that processes such as apoptosis caused by treatment are accompanied by the secretion of cytokines, growth factors, and microRNAs (68). As a result, certain studies are focused on exploring whether microRNAs can function as biological indicators for evaluating the efficacy of treatments.

#### 3.2.1 Radiofrequency therapy and microRNA

Radiofrequency ablation is a safe and effective treatment option for patients with early-stage, unresectable, and non-transplantable HCC nodules that are less than 3 cm in diameter (69). Studies comparing local radiofrequency therapy and surgical resection have found no significant difference in progression-free survival (RFS) or overall survival (OS) between the two treatments (70). However, a significant proportion of patients experience tumor recurrence (71). To effectively monitor recurrence after radiofrequency therapy, it is crucial to have a non-invasive biomarker. The Let-7 family, as previously mentioned, is particularly useful for detecting HCC in its early stages, particularly in patients with risk factors for hepatitis C infection. Let-7c is a member of the let-7 family that has been found to have a cancer-suppressing effect. It achieves this by inhibiting tumor cell proliferation and promoting apoptosis. This is achieved through its ability to target CD25A, PI3K/Akt/FoxO, and Wnt signaling (72, 73). It is worth noting that Let-7c is down-regulated in HCC tissues. Research has demonstrated that the levels of circulating let-7 can indicate early recurrence following treatment for HCC. Additionally, overexpression of let-7 has been linked to portal vein invasion, changes in TNM stage, and overall survival post-treatment (74). Similarly, studies have shown that patients treated with local radiofrequency ablation and who have elevated levels of circulating let-7 are more likely to experience a recurrence of HCC after treatment (71). Radiofrequency therapy primarily relies on the direct cautery of tumor tissue through high temperatures. As a result, the peak of circulating microRNA typically occurs approximately 60-90 minutes after the radiofrequency treatment (75). While let-7 is known to act as a tumor suppressor during tumorigenesis, recent findings suggest that microRNAs may have varying roles, and even exhibit opposing patterns, within the tumor environment. Although the exact mechanism remains unclear, it is possible that circulating blood cell secretion may be involved. In a study of patients who underwent radiofrequency therapy, it was found that levels of circulating miRNA-210 and miRNA-200a increased in those who experienced early progression (75). MiRNA-210 has been shown to play a role in tumor progression and metastasis by regulating various cellular processes such as mitochondrial metabolism, angiogenesis, cell proliferation, and apoptosis (76). The function of miRNA-200a in tumorigenesis and progression remains unclear, although it is widely believed to act as a tumor suppressor. Evidence suggests that elevated levels of miRNA-200a in circulation are associated with a negative prognosis in patients with colorectal cancer (77). Clinicians can improve their assessment of a patient’s treatment by analyzing the fluctuations in microRNA levels present in circulation. This allows for the adjustment of treatment strategies that are appropriate for the patient’s needs.

#### 3.2.2 Transarterial chemoembolization therapy and microRNA

For patients with advanced stages of cancer and larger tumors, transarterial chemoembolization is a highly recommended treatment option (78). This treatment primarily involves injecting chemotherapy drugs, such as cisplatin, into the arteries. This is then followed by the embolization of the tumor’s corresponding blood vessels using embolic agents (79). Under high concentrations of embolism, tumor cells experience hypoxia and a cytostatic effect. Certain tumors may not respond to arterial injection of cisplatin, which highlights the importance of identifying a reliable biomarker that can effectively assess the effectiveness of treatment. The study revealed that patients who responded well to TACE had higher baseline levels of miR-106b, miR-107, and miR-133b compared to non-responders. On the other hand, it was found that non-responders exhibited elevated levels of miR-26a and miR-31 compared to responders. Notably, miR-133b and miR-26a demonstrated the most promising response to TACE treatment, with miR-133b proving particularly effective in distinguishing between complete response, partial response, and no response (80). In other tumors, miR-133b has been discovered to increase sensitivity to chemotherapy drugs by inhibiting ABCC1 and MDR1. Additionally, it has been identified as a tumor suppressor factor (81, 82). Researchers have discovered that combining miR-26a with other microRNAs can accurately predict the effectiveness of treatment within one year after TACE surgery for HCC (83). Following TACE treatment, the levels of circulating microRNA will reach their highest point after 24 hours. This is due to the fact that TACE treatment is a slower ischemic process, which means that it takes longer to reach its peak compared to radiofrequency therapy (75). TACE therapy has been found to increase the levels of miRNA-210 in circulation. However, there is a lack of studies that have investigated the correlation between this increase and patient prognosis.

#### 3.2.3 Chemotherapy and microRNA

Chemotherapy drugs for HCC are typically categorized into three main types: chemotherapy, targeted therapy, and immunotherapy. While immunotherapy drugs have been approved for widespread clinical use (84), they may not be suitable for patients with poor liver function or a history of autoimmune diseases. In such cases, targeted drugs like sorafenib are the optimal solution for drug treatment. One of the primary challenges in using sorafenib for clinical purposes is the development of drug resistance that occurs during the course of treatment. If clinicians can identify a biomarker that indicates resistance to therapeutic drugs, it could greatly improve the prognosis of patients receiving advanced drug therapy. Serum exosomes found in patients with HCC have been found to inhibit apoptosis through the HGF/c-Met/Akt pathway. This leads to resistance to sorafenib, a common treatment for HCC (85). This demonstrates that circulating microRNAs play a role in the development of drug resistance. Studies have demonstrated that miR-31-5p, miR-221, miR-30e-3p, and miR-30d are linked to sorafenib resistance in hepatoma cells that are cultured *in vitro (* 86, 87). High levels of miRNA-30E-3p in the bloodstream are frequently indicative of the onset of sorafenib resistance. However, the precise mechanism behind this phenomenon remains unclear. It is possible that sorafenib prompts TP53 to enhance extracytocrine, which could be a contributing factor. Targeting MDM2 primarily results in inhibiting tumor growth when TP53 is present, whereas targeting PTEN and p27 under TP53 regulation promotes tumor development (87). High levels of miRNA-518d-5p in serum correlate with survival time during treatment with sorafenib (61). The process in question may be linked to PUMA, which is the primary regulator involved in the apoptosis process. PUMA is mainly expressed in the mitochondria and works in conjunction with sorafenib to enhance tumor suppression (88). A clinical cohort study on sorafenib treatment revealed that miR-200c-3p serum levels were upregulated post-treatment. Furthermore, the study found that patients with HCC who had elevated levels of miR-200c-3p had a lower mortality rate. After sorafenib treatment, both miR-222-5p and miR-512-3p levels in circulation were found to be downregulated. Furthermore, the clinical survival data indicated that patients with a decreased level had a poorer outcome (89). Elevated levels of miRNA-10-3p in serum suggest that sorafenib treatment may be effective, while higher concentrations indicate that the drug is actively impacting tumor cells. This is supported by the fact that Cyclin E1, a promoter associated with sorafenib resistance, is a downstream target of miRNA-10-3p. These findings underscore the clinical relevance of miRNA-10-3p as a biomarker for predicting the efficacy of sorafenib treatment (90). By analyzing these serum markers, clinicians are able to assess the effectiveness of a patient’s drug treatment and ultimately enhance their prognosis.

## 4 Long non-coding RNA

Long noncoding RNA (lncRNA) is a type of linear RNA that exceeds 200 nucleotides in length. Unlike other RNA molecules, lncRNA does not encode proteins or peptides. However, its secondary and three-dimensional structure allows it to perform functions similar to both RNA and proteins (91). LncRNA is primarily found in the nucleus (92), but it can also operate in body fluids by being secreted through exosomes (93).

In comparison to microRNAs, lncRNAs exhibit a wider range of behavioral patterns, which have been validated in both pathophysiological and pathological contexts. It interferes with mRNA editing or other independent signaling pathways, affecting the transcription and translation of genes. (LncRNAs) have various functions, which can be categorized into three main types: cis-action, trans-action, and regulation of adjacent gene expression. Cis-acting LncRNAs can either activate or inhibit chromatin. Meanwhile, LncRNAs can affect the expression of adjacent genes by modulating the location of RNA polymerase II (94). It has been established that dysregulated lncRNAs can play a role in tumor formation by affecting various cellular processes, such as cell proliferation, migration, invasion, epithelial to mesenchymal transformation, apoptosis, and anti-tumor resistance (95, 96). Currently, there are studies that examine the relationship between the level of detection in circulation and the development and spread of tumors. Specifically, lncRNA has been found to play a role in the occurrence and development of hepatocellular carcinoma. Furthermore, early changes in lncRNA levels in circulation can serve as targets for early detection of tumors (97). LncRNA is utilized as a biomarker for tumors, which improves the sensitivity of diagnosing HCC and the prognosis of tumors.

### 4.1 lncRNA and HCC diagnosis

The lncRNA molecules NBAT-1 and FOXCUT have been found to have increased serum levels in patients with hepatitis C virus-induced hepatocellular carcinoma (HCC). Furthermore, higher levels of these molecules appear to be associated with more positive clinical outcomes in patients with HCC (98). The NBAT-1 gene is known to suppress tumors and is typically down-regulated in various types of cancer. Its primary function is to hinder cell proliferation and migration by controlling cancer-causing microRNAs and transcription factors (99). The reason for the upregulation of NBAT-1 in the serum of patients with HCC is primarily the body’s natural defense mechanism against the growth of tumors. FOXCUT is a tumor suppressor gene that plays a crucial role in inhibiting cell proliferation, migration, and invasion. It also induces cell cycle arrest and apoptosis. Moreover, the induction of FOXCUT significantly reduces the expression of matrix metalloproteinases (MMPs). These endopeptides are responsible for mediating extracellular matrix degradation, which promotes tumor growth (100). Based on the collation of data from the database, researchers have identified three lncRNAs, namely AC005332.5, ELF3-AS1, and LINC00665, that can serve as effective markers for the diagnosis of hepatocellular carcinoma. These lncRNAs have demonstrated promising responses in the model (101). In patients with HCC, the serum level of lncRNA SCARNA10 was found to be significantly higher than that in both the benign liver disease and control groups. Furthermore, there was a positive correlation between the level of lncRNA SCARNA10 and the degree of tumor malignancy. When combined with AFP, the ROC curve showed higher sensitivity (102). The biomarkers MIR4435-2HG and lnc-POLD3-2, as well as their combinations, have been demonstrated to be effective in distinguishing between hepatocellular carcinoma and non-hepatocellular carcinoma. The ROC curves indicate that these biomarkers have higher sensitivity in patients with normal alpha-fetoprotein levels (103). In a recent study (104), it was demonstrated that circulating lncRNA is consistent with the tumor microenvironment, and that levels of circulating lncRNA can reflect immune infiltration within tumors. The study also found that the expression of MIR4435-2HG and lnc-POLD3-2 in circulation could potentially serve as biomarkers for HCC (103). The lncRNA MIR4435-2HG plays a role in the induction of HCC through the WNT/β-catenin and transforming growth factor-β pathways (105). Its upregulation in serum levels is indicative of poor tumor biological behavior. The lncRNA HOTAIR (homeobox transcript antisense intergenic RNA) plays a crucial role in regulating epigenetic modifications among multiple genes. It is also implicated in the metastasis and drug resistance of various tumors (106). The lncRNA BRM (associated with Brahma) and lncRNA ICR (long noncoding RNA related to ICAM-1) are both found to be highly expressed in liver malignancies (107). Elevated levels of serum lncRNA HOTAIR, BRM, and ICR have been identified as accurate diagnostic markers for hepatocellular carcinoma (108). In patients with HCC, the concentration of LINC01793 in whole blood was significantly higher compared to healthy individuals or those with chronic hepatitis. The diagnostic efficacy was found to be highest when combined with AFP (109). The lncRNA cancer susceptibility candidate gene 2 (CASC2) acts as a tumor suppressor gene in endometrial cancer by being expressed in a down-regulated manner. On the other hand, the lncRNA taurine up-regulated gene 1 (TUG1) has been found to contribute to tumor formation in various types of cancers. In patients with hepatitis C-induced HCC, the expression of CASC2 was found to decrease, while the expression of TUG1 increased in healthy individuals (110). This suggests a potential antagonistic relationship between the two, although the exact mechanism remains unclear. The NEAT1 lncRNA is a significant constituent of parameningeal proteins and has been linked to various types of cancer. The analysis of the TCGA database revealed that NEAT1 is expressed at high levels in patients with hepatitis C virus-induced HCC. Furthermore, there was no significant decrease in serum NEAT1 levels after antiviral treatment (111). This suggests that NEAT1 may play a role in triggering liver malignancy and could serve as a useful biomarker for early HCC diagnosis.

### 4.2 lncRNA and HCC prognosis

In both HCC stem cells and mice, the expression of lncRNA-H19 increased as the disease progressed. The researchers also discovered that patients who exhibited high levels of lncRNA-H19 after treatment often experienced tumor recurrence (112). In their study, Huang et al. found that a recently identified lncRNA, RP11-85G21.1 (Lnc85), plays a significant role in promoting the proliferation and migration of hepatoma cells. This is achieved through its binding and regulation of miR-324-5p, ultimately impacting patient prognosis. The lncRNA CRNDE has been shown to increase the migration, activity, and invasion of hepatoma cells (113). In this study, we found that the expression level of lncRNA CRNDE in serum is positively correlated with tumor volume and negatively correlated with degree of differentiation. Furthermore, our analysis revealed that lncRNA CRNDE is an independent predictor of overall survival time in patients with HCC. Including lncRNA CRNDE as a marker can accurately predict the prognosis of HCC (114). LINC00161 is an oncogene that is involved in promoting the migration and invasion of hepatocellular carcinoma (HCC). LINC00161 has been identified as a biomarker for HCC in various studies. It has been found to have elevated levels in the serum of HCC patients. Additionally, LINC00161 has been linked to lower survival rates in HCC patients (115).

## 5 Conclusions and perspectives

Hepatocellular carcinoma is the primary form of liver cancer, with hepatitis viruses - particularly hepatitis B and C - being endemic in Asia. This has resulted in a high incidence and mortality rate of HCC in the region. Non-alcoholic fatty cirrhosis and alcoholic cirrhosis are both considered high-risk factors for HCC. The onset of HCC is often insidious, and the tumor is frequently discovered in its early stages. However, by the time a liver mass lesion is identified, the tumor may have already advanced and spread to other areas, such as distant metastasis or portal vein cancer thrombus. There is an urgent need for an effective biological marker that can detect and diagnose hepatocellular carcinoma in its early stages. Currently, the diagnostic criteria for HCC rely on imaging combined with serum marker AFP. However, imaging is not very effective in detecting early lesions, and the specificity and sensitivity of serum AFP are low. As a result, it is imperative to establish a more dependable diagnostic approach. Recent experiments have demonstrated the involvement of non-coding RNA in tumor development and progression. Additionally, non-coding RNA can be released into the bloodstream via tumor cell death or the circulation of exosomes containing tumor and blood cells. Thus, the significance of non-coding RNA in cancer diagnosis cannot be overstated. The non-invasive extraction of biomarkers is commonly referred to as “liquid biopsy”. In recent times, numerous studies have demonstrated the effectiveness of circulating microRNAs (such as miRNA-122, let-7 family, miRNA-34a, and other circulating microRNAs) and lncRNA in the early detection of HCC (**Table 1**). Additionally, many studies have suggested that combining circulating non-coding RNA with serum AFP can lead to even higher diagnostic efficiency. In the treatment of HCC, surgical radical resection and liver transplantation are considered the most effective methods. However, it is important to note that surgical treatment may have limited benefits for patients with advanced HCC. This has led to the development of alternative treatment options such as transarterial chemoembolization, radiofrequency ablation, and drug therapy for patients with HCC. Currently, targeted therapy is the primary drug treatment for HCC. However, the use of drugs can lead to the development of drug resistance in tumors. Prior research has examined the alteration of serum noncoding RNA levels in patients before and after surgery. By utilizing a specific biomarker to assess the patient’s condition following treatment, the prognosis for HCC treatment can be enhanced. In the context of TACE and radiofrequency therapy, circulating microRNA has been identified as a crucial factor in treatment evaluation and early postoperative recurrence. Similarly, in patients receiving sorafenib, certain molecules such as miR-31-5p, miR-221, miR-30e-3p, and miR-30 in circulation have been found to indicate drug resistance over time. Additionally, doctors can use lncRNA in serum to determine the prognosis of patients. In summary, HCC patients have a higher number of non-coding RNAs (ncRNAs) which are highly sensitive to HCC. These ncRNAs are secreted into the bloodstream, providing clinicians with valuable information about tumors in a minimally invasive manner. If this technology is applied in clinical settings, it has the potential to revolutionize the treatment strategy of HCC and significantly improve patient prognosis.

**Table 1:** Circulating ncRNA and HCC.

Circulating ncRNA	Expression	Pathway	Function	Reference
miRNA-122	up-regulated	Cyclin G1/insulin-like growth factor 1 receptor	Diagnosis	(30)
let-7	down-regulated up-regulated	IL-10 CD25A, PI3K/Akt/FoxO and Wnt signals	Diagnosis Efficacy assessment	(47) (71, 72),
miRNA-34a	down-regulated	Transcription activators E2F1/E2F3, caspase-3	Diagnosis	(51, 52),
miRNA-193a-5p	up-regulated	Nucleoli and spindle-associated protein 1	Diagnosis	(57)
miRNA-223-3p	down-regulated	insulin-like growth factor-1 signaling pathway	Diagnosis	(59)
miRNA-16	up-regulated	SETD3	Diagnosis	(41)
miRNA-518d-5p	up-regulated	–	Diagnosis	(60)
miRNA-107	up-regulated	–	Diagnosis	(61)
miRNA-210	up-regulated	mitochondrial metabolism	Efficacy assessment	(75)
miRNA-200a	up-regulated	–	Efficacy assessment	(76)
miR-133b	up-regulated	ABCC1/MDR1	Efficacy assessment	(80, 81),
miR-26a	down-regulated	–	Efficacy assessment	(79)
miRNA-30E-3p	up-regulated	TP53	Efficacy assessment	(86)
miRNA-518d-5p	up-regulated	PUMA	Efficacy assessment	(87)
miR-222-5p	down-regulated	–	Efficacy assessment	(88)
miRNA-10-3p	up-regulated	Cyclin E1	Efficacy assessment	(89)
FOXCUT	up-regulated	MMPs	Diagnosis	(96)
SCARNA10	up-regulated	–	Diagnosis	(100)
MIR4435-2HG	up-regulated	WNT/β-catenin TGF-β pathways	Diagnosis	(103)
HOTAIR	up-regulated	Epigenetic modifications	Diagnosis	(105)
CASC2	up-regulated	–	Diagnosis	(108)
RP11-85G21.1	up-regulated	miR-324-5p	Prognosis	(111)
CRNDE	up-regulated	–	Prognosis	(112)

## Author contributions

All authors participated in conception and design. JY wrote the manuscript. YX and YC revised the manuscript. All authors read and approved the final manuscript.
